# Miscellaneous Intelligence

**Published:** 1820-01-01

**Authors:** 


### Miscellaneous Intelligence.

" *Extract of a Letter from* W. Jardine, Esq. *Dumfries.*

" A poor boy having swallowed a double pike-hook, has been a long time under the care of the Gentlemen of the Royal Infirmary, Edinburgh, but without any account having been laid before the public by them, relative to the extraction.

" As soon as I heard of the accident, I sent the following description of an instrument adapted to the purpose of extraction.

" This instrument is in the form of a common probang, with a conical sponge, perforated in the centre, about three-quarters of an inch in diameter at the top, and as thick near its base, as can be pushed into the œsophagus, so thick, that its semi-diameter may considerably exceed the breadth of the hook.

" In using this instrument, the chain of the hook hanging on the outside of the mouth, is first to be put through the hole in the sponge; the sponge is then, by means of the chain, to be directed down to the hook, and if, in the dilatation of the œsophagus by the sponge, the hook is not unfixed, the instrument must be pushed on a little farther, or until it draws in the chain, when we may presume the hook is disengaged; the chain then, in being drawn gently up, will direct the points of the hook, in which ever way the hook is turned, into the sponge, and so prevent them from catching any thing in their way up, when the instrument and chain are withdrawn."

The hook is said to have been extracted by an instrument differing from the above in having an ivory spheroid in place of the sponge, which was perforated oblongly, as the sponge, and directed by the chain to the hook. Mr. Jardine, whose ingenuity in the construction of instruments is well known, makes many forcible objections to the ivory spheroid, particularly its unyielding hardness. For, although by directing it against the curves of the hook, when slightly attached by the points, we may disengage them, yet, we cannot so easily do so, if held by the barbs, since the force necessary for this purpose will stretch the œsophagus so much, as to transfer the pressure entirely to the points, which may drive them through the coats of the œsophagus, and bring them in contact with the spheroid; consequently drive the barbs farther in. The sponge is free from these and many other objections. It is probable, from the facility with which the hook is said to have been extracted from the boy's throat, either that the points entered very slightly, or that a suppuration, as the writer in the Carlisle Journal supposes, had taken place.

Newcastle under Lyme, Staffordshire.
July 21, 1819.

Sir,

I have now before me your paper on Phlegmatia Dolens in Dr. Johnson's Journal for July, wherein you seem to express a doubt of the disease attacking the same limb twice.

I have at present among my patients, a lady, who has borne four children, and has experienced three violent attacks of this disease; viz. after the birth of the first, third, and fourth child, her labours having always been easy and natural, and her general health good, excepting a decided tendency to constipation, which has required great attention during the periods of gestation.

The first attack commenced with pain in the right groin, which gradually extended down the thigh and leg, till it embraced the whole limb. The second commenced in the left calf, and extended upwards to the groin; this attack was very acute, and long protracted. I had assured her that she would have no more returns, but the third was the severest of all, commencing about four days after parturition again in the right groin, and after violently afflicting the thigh and leg, fixed itself with equal, if not greater severity, in the left limb. They have left no lameness nor enlargement, if you except a tendency to swell in the evening, and a feeling of stiffness upon increased exertion.

A liberal use of mercurial purgatives, besides the usual topical applications, was what I principally trusted to, the alvine evacuations being throughout fœtid and bilious, and the disease certainly yielding as they returned to a healthier state. You will excuse these few observations, from one, who, though unknown to you, is a sincere admirer of your talent and zeal for medical improvement; if they are worth your attention, I may return to the subject.

I remain,

Your obedient Servant,

H. S. Belcombe, M. D.

To Dr. Dickson, F. R. S. E.

&c. Clifton.

P. S. By an error of the Press, *Phlegmatia*, was printed *Phlegmasia*, throughout Dr. Dickson's paper. Ed.

---

Mr. Dunglison, of Prescott Street, has lately laid an interesting paper on the use of Moxa, as an article of French Therapeutics, before the Hunterian Society. The following case was shewn to the author by Baron Larrey, at the hospital of Gros Caillon, in the beginning of 1818.—" A cavalry soldier, aged 22 years, was admitted into the aforesaid hospital, in December 1814, labouring under all the symptoms of hip disease of the right side, in its second stage, with an abscess situated on the outward and anterior part of the joint. This tumour was elevated about an inch above the neighbouring parts, and was about three inches in diameter. The diseased extremity, which could scarcely endure the least motion, when placed along side the other, appeared to be about an inch longer. The patient was cupped many times, previous to the application of the Moxa; and until the fifth time, no evident effect appeared to be produced by this mode of treatment. But after the eighth and ninth application of the Moxa, the tumour was reduced one quarter. By successive applications of the Moxa, the tumour diminished; but Baron Larrey being under the necessity of leaving Paris, the care of the patient devolved on Dr. Pigou, who pursued the same mode of treatment until the patient was completely cured. When Mr. Dunglison saw this man, with M. Larrey, he appeared very little lame, and the limb but triflingly deformed, being about half an inch shorter than the other."

---

*Rules and Regulations of the Associated Apothecaries and Surgeon-Apothecaries of England and Wales, unanimously approved, and adopted, at the Annual General Meeting of the Association; held, by public Advertisement, at the Crown and Anchor Tavern, Strand, London, July* 21, 1819.

### SECT. I. OF ITS NAME.

This Association shall in future be called, "*The Associated Apothecaries and Surgeon Apothecaries of England and Wales.*"

### SECT. II. OF ITS OBJECTS.

ART. I. It is constituted to promote the improvement of those branches of the profession exercised by general practitioners, and by thus supporting their respectability, to protect, in the most effectual manner, the interests of the community at large.

II. To ensure this respectability, by embracing every opportunity of securing to the practice of surgery, and of midwifery, similar regulations to those which now legally control the practice of medicine.

III. To endeavour to prevent ignorant and unqualified persons from practising medicine, by watching over the operations of the apothecaries' act, and by communicating to the society of apothecaries all cases in which it shall be discovered, that the Regulations of that act shall have been infringed.

IV. To establish such communication between the committee and country members, as may facilitate the transmission of useful suggestions and facts; and furnish them with such conclusive evidence, as may be serviceable in any future application to parliament.

V. To form and preserve lists of practitioners legally appointed; and thus, by distinctly separating the two classes, to enable the public, in some degree, to distinguish the fair and authorized practitioner, from the ignorant unqualified pretender.

### SECT. III. OF MEMBERS.

I. This association shall consist of the hereinafter-mentioned gentlemen, subscribers to the original association.

II. The requisite qualification for the admission of every future member shall be, that he is legally authorized to act as an apothecary or a surgeon.

III. Before admission, a certificate testifying his qualifications, signed by two members of this association, and accompanied by a declaration of the candidate's wish to become a member, shall be transmitted to the chairman of the metropolitan general committee, or to the chairman of the county general committee, as the case may be, at least one fortnight before the ordinary meetings of such committee; and the election shall take place at the next quarterly meeting of the association.

IV. The fee for admission shall be one guinea, to be paid on admission.

V. That all members present at the determination of any question, shall possess an equal right to vote.

VI. That honorary members be nominated and appointed only at the general meetings of the association.

### Sect. IV. OF MEETINGS.

I. The association shall meet for general purposes once in each year, on the third Wednesday in June.

II. One fortnight's notice shall be given of the annual general meeting, in the London Medical Journals, and by advertisements in two morning and two evening papers.

III. The association shall, on that occasion, assemble at one o'clock in the afternoon. The president shall take the chair for the dispatch of business precisely at two o'clock, and the business shall be conducted in the following order; viz.

Candidates proposed.

Candidates balloted for.

Minutes of the preceding meeting read, and discussed.

Report of the general committee received, read, and considered.

Miscellaneous business, including the revisal of the laws, and whatever may relate to the general management of the association.

IV. No question relative to the alteration of any fundamental laws of the association shall be discussed, unless notice of it, signed by at least three members, shall have been transmitted to the president, at least three months before the annual meeting of the association, so as to allow time for country members being duly apprized of such intention.

V. All questions on which a difference of opinion may exist, shall be decided by a show of hands, or division, at the option of the members present.

VI. In cases where the suffrages are equal, the president shall have a casting vote.

VII. After the conclusion of the business of the general meeting, the members shall dine together, at five o'clock.

VIII. Special general meetings of the association may be called by the president, by virtue of his office; and shall be summoned by him, on receiving a requisition signed by five members of the general committee, or by fifteen members of the association, the proposed object of such meeting being announced to the members in each case.

IX. Special general meetings of the association, shall be subject to all the foregoing regulations, except Article VII.

X. Members residing in the country shall not only have the right of attending all the meetings of the association, but their presence will always be considered as desirable, particularly at the annual general meeting.

### Sect. V. OF COMMITTEES.

I. A general committee shall be formed, to whom shall be entrusted the conducting of the business of this association, between its general meetings, according to the existing laws.

II. The general committee shall consist of the president of the association, two vice-presidents, and twenty-four members, twelve of whom shall be chosen from such members of this association as do, and twelve from such as do not belong to the apothecary's society.

III. The president shall be chairman of the general committee, and have a casting vote.

IV. One half of each class of the committee shall go out every year by rotation; such members shall nevertheless be eligible for re-election.

V. The general committee shall meet quarterly for dispatch of business; viz. on the first Monday in September, December, March, and June.

VI. Meetings of the general committee may be called by the chairman, by virtue of his office, and shall be summoned by him, on receiving a requisition, signed by five members of the committee

VII. The general committee shall appoint a sub committee, out of its own members, whenever it shall be deemed necessary.

VIII. At meetings of the general committee, five members shall form a quorum.

IX. At meetings of the sub-committee, three members shall form a quorum.

X. The general committee shall present a report of its proceedings during the last year, to the association, at its annual meeting in June.

XI. The members of this association, who reside in the country, shall form themselves into county committees.

XII. Each county committee, when duly organized, shall form sub committees for as many districts as the extent and population of the county may, after mature consideration, appear to be requisite.

XIII. The county and district committees shall choose, from their own bodies, respectively, a chairman and a secretary.

XIV. The meetings of the county and district commitees shall be held at periods, and places, most convenient to the members of such county or district.

XV. A general meeting of the members in each county shall be held every year, at least one month previous to the annual general meeting of the association in the metropolis; the day, and the place of meeting to be determined by the county committee.

XVI. The business of the annual county meeting shall be, to receive communications from the several district committees; to receive application for the admission of members: and to transmit the same to the metropolitan committee, with such other information, and such suggestions, as may appear to be of sufficient importance to require the attention of the general meeting.

XVII. The objects of the county and district committees shall be; to take cognizance of, and communicate, any instances of infringement upon the regulations of the apothecaries' act in their respctive neighbourhoods; and to inquire into all cases of gross ignorance, or impropriety of conduct; and to transmit the same, through their chairman, (when duly authenticated) to the chairman of the metropolitan committee for the use of the association; and, if necessary, for further transmission to the society of apothecaries.

### Sect. VI. OF OFFICERS.

I. The officers of the association shall consist of a president, two vice presidents, three treasurers, and a secretary.

II. The president and vice presidents shall be chosen annually.

III. There shall be two vice presidents, one only of whom shall be chosen from the apothecaries' society.

### Sect. VII. OF THE PRESIDENT.

It shall be the province of the president to preside at the meetings of the association, and to enforce an observance of its rules.

### Sect. VIII. OF THE VICE-PRESIDENTS.

In the absence of the president, his duties shall devolve upon one of the vice-presidents present.

### Sect. IX. OF THE TREASURERS.

I. It shall be the duty of the treasurers, to invest and keep in public securities, in their joint names, all monies belonging to the association, (except such balance as shall be necessary to defray current expenses) for the exclusive use and benefit of the association.

II. The signature of two of the treasurers shall be necessary, to the disposal of any part of the funds of the association.

### Sect. X. OF THE SECRETARY.

It shall be the office of the secretary, to attend the several meetings of the association in London; to enter on the journals the minutes of the proceedings of such meetings; to keep a printed list of the members of the association; and to act under the general direction of the president.

Mr. Curtis will commence his Winter Course of Lectures on the Anatomy, Physiology, and Diseases of the Ear, early this Month, at the Royal Dispensary for Diseases of the Ear, Carlisle Street.

A Medical Review, on a more extensive scale, (an Octavo Volume Quarterly) but much on the same plan as this Journal, has just commenced in Paris, and promises to be of great benefit to Medical Science. We have put ourselves in direct communication with Dr. Rouzet, the managing Editor, and a mutual exchange of Journals will regularly take place. In our next Number we shall exhibit a comprehensive analysis of this interesting foreign cotemporary; and hope, through it, to enrich, from time to time, the Medical and Surgical Literature of the British Empire and Dependancies.

In the ensuing month will be published, A Defence of the Scriptural Doctrine of Life, as applied to Physiology and Medicine; being a Reply to the Remarks of certain Reviewers, on a recent Work of the same Author, in which that Doctrine is advocated. By John G. Mansford, Member of the Royal College of Surgeons, and Surgeon at Bath.

Dr. A. P. Wilson Philip has in the Press a Fourth Edition of his Treatise on Fevers, in one volume 8vo. including the various Species of Simple and Eruptive Fevers.

---

The unprecedented sale of the last number of our Journal, and the unanimous approbation which has been expressed of its plan, have determined us to adhere strictly to the course of "*Analytical Reviews*," as the channel through which we can convey the greatest quantum of useful matter in the shortest space: and, since the public approbation has conferred a degree of importance on this Journal which we never anticipated, so, in gratitude for this, we are determined to henceforth develope new energy and exertion, in rendering it worthy of such distinguished patronage.

The Subscribers will perceive the increase of labour and expence which we have imposed on ourselves in the present number of the Journal, by printing nearly half of it on a type which has enabled us to concentrate an immense *additional* quantum of important information; besides which, twelve extra pages are appended to this Number.

Notwithstanding all our exertions, we have been obliged reluctantly to postpone several important reviews, especially of the Bombay Medical Board's Report of the Indian Epidemic; Hall on the Mimoses; Hunter on Mineral Waters; Simpson on Hemeralopia, Cheyne on Hydrocephalus Internus, Jackson on Contagious Fever, &c. all of which are ready for the Press, and are only delayed for want of room.

Communications have been received from Dr. Stowe, of Buckingham; Mr. George Bullen, of Ipswich; Mr. Macewan, of Grenada; Mr. Fitzpatrick, of the Royal Artillery; Dr. Hamet, of the Royal Navy, (through Dr. George Pearson); Mr. W. S. G. Davis, Surgeon, Broad-street, Golden-square; Mr. Montgomery, Paris; Dr. W. A.

